# Machine learning for predicting surgical difficulty of laparoscopic total mesorectal excision for rectal cancer: integrating MR-based pelvimetry and peritoneal reflection

**DOI:** 10.3389/fmed.2026.1853753

**Published:** 2026-06-12

**Authors:** Shaoting Zhang, Fangying Chen, Minglu Liu, Yukun Chen, Guodong Jing, Xiaolu Ma, Chengwei Shao, Xiaohui Shi

**Affiliations:** 1 Department of Radiology, Changhai Hospital, Naval Medical University, Shanghai, China; 2 Department of Radiology, Shandong Cancer Hospital and Institute, Shandong First Medical University and Shandong Academy of Medical Sciences, Jinan, China; 3 Department of Colorectal Surgery, Changhai Hospital, Shanghai, China

**Keywords:** machine learning, pelvimetry, peritoneal reflection, rectal cancer, surgical difficulty

## Abstract

**Background:**

To investigate the potential of a machine learning (ML) model integrating MR-based pelvimetry and the peritoneal reflection (PR) in predicting the surgical difficulty of laparoscopic total mesorectal excision (LaTME) for rectal cancer (RC).

**Methods:**

RC patients who underwent rectal magnetic resonance imaging (MRI) before LaTME between December 2020 and December 2022 were included in this retrospective study. The duration of surgery, blood loss, postoperative hospital stay, and postoperative complications were selected to evaluate the surgical difficulty. Using unsupervised clustering analysis, all patients were divided into surgical difficulty group and non-surgical difficulty group based on four indicators of surgical difficulty. Least absolute shrinkage and selection operator (LASSO) regression and logistic regression were used to identify factors influencing the surgical difficulty of LaTME, and logistic regression (LR) and the extreme-gradient boosting (XGB) models were constructed. The receiver operating characteristic (ROC) curve and decision curve analysis (DCA) were utilized to compare the performance of the two models.

**Results:**

Data from 283 patients (60.98 ± 11.17 years old, 204 males and 79 females) were evaluated. The LASSO regression suggested that pelvic depth, the distance from the lower tumor margin to PR (T-PR), and gender were associated with the operative difficulty. The XGB model with an area under curve (AUC) of 0.809 (95% CI: 0.757–0.862) demonstrated a better performance than the LR model with an AUC of 0.623 (95%CI: 0.553–0.694). DCA confirmed that the XGB model was superior to the LR approach.

**Conclusion:**

The ML model integrating the pelvic depth, T-PR, and gender can evaluate the surgical difficulty of LaTME preoperatively and non-invasively, thus facilitating the predictive, preventive, and personalized treatment process in RC treatment.

## Background

Rectal cancer (RC) constitutes a relevant public health challenge worldwide, making up 30–35% of all colorectal cancer (CRC) cases (1, 2). Surgery remains the most effective treatment for RC, with the standard procedure reducing local recurrence and positive circumferential margin rates (3, 4). In addition, the quality of the surgery directly affects local tumor control and prognosis (5). In recent years, laparoscopic total mesorectal excision (LaTME) has been broadly applied in RC treatment as an effective and safe procedure. Compared with open surgery, LaTME confers short-term benefits regarding complications, postoperative hospitalization duration, and mortality (4, 6), and it can significantly reduce bleeding during operation (7). However, not all patients administered LaTME have high-quality rectal TME surgery, it remains controversial for mid- or low-rectal cancer (7). Thus, preoperative prediction of surgical difficulty in LaTME is critical. Patients should be provided with a more objective understanding of the overall surgical procedure and difficulty beforehand; in addition, surgeons should design the overall surgical plan based on predicted operative difficulty to minimize the occurrence of intraoperative accidents.

Several studies have highlighted the importance of predicting surgical difficulty for achieving better surgical outcomes. Variables such as body mass index (BMI), gender, tumor location and pelvimetry measurements by magnetic resonance imaging (MRI) are known risk factors associated with surgical difficulty related to TME (5, 8–10). However, there are some inconsistencies between studies. For example, some reports have found no association between MRI pelvimetry and surgical difficulty (8–11). Pelvimetry data processing is often complex and time-consuming, which may be a possible reason for this discrepancy. The current prediction involves a subjective process lacking inter-rater reliability in clinical settings. Another reason is that the definition of tumor location differs according to the classification method. According to the NCCN guidelines, tumor locations are divided into upper, middle, and lower groups (10–15, 5–10, and < 5 cm, respectively) based on the distance from the distal end of the tumor to the anal verge (AV) (12). Meanwhile, according to the location relationship between tumor and peritoneal reflection (PR), tumor locations are assigned to three positions based on MRI imaging: ➀above the PR, ➁straddling the PR, and ➂below the PR (13). Therefore, the lack of unified pelvimetry measurements and understanding of the definition of tumor location might be the potential reason for the limited application of surgical difficulty prediction. Our previous study revealed that MRI could identify PR to evaluate the distance from the inferior tumor margin to PR (T-PR) and tumor location with regard to PR (L-PR); the consistency between observers and the accuracy of determining tumor location was relatively high (13). Meanwhile, Gao et al. found that L-PR evaluation might be beneficial for prognosis and treatment planning in RC patients (14). Thus, PR is a valuable marker for identifying tumor location, which helps clinicians choose treatment options (15–17). However, so far, no studies have assessed factors associated with PR for validating the short-term perioperative outcomes of LaTME in RC patients.

With the development and broad application of artificial intelligence (AI), machine learning (ML) has been widely applied in oncology. For example, ML has been used for rectal lesion identification, T-stage, and lymph node metastasis evaluation, prediction of treatment outcome and survival, and assisting individualized diagnosis and treatment (18–22). In addition, extreme-gradient boosting (XGB), a machine learning algorithm that works based on gradient-boosted decision trees, has been used for disease diagnosis, risk prediction, outcome prediction, prognosis, drug use, drug research and development in the medical field (23, 24). However, its application in estimating technical difficulties in performing laparoscopic rectal surgery for RC is limited. Therefore, in this study, we aimed to investigate the potential of an ML model integrating MR-based pelvimetry and PR in predicting the surgical difficulty of LaTME for RC.

## Materials and methods

### Patients

All methods used in the present research were conducted in accordance with the Declaration of Helsinki and were approved by the local Institutional Review Board (Committee on Ethics of Biomedicine of Changhai Hospital). Informed consent was waived for this retrospective study. Patients with RC scheduled for LaTME and who underwent preoperative rectal MRI between December 2020 and December 2022 were enrolled from the Colorectal Surgery Department of Changhai Hospital (a tertiary referral center specializing in the treatment of colorectal cancer).

**FIGURE 1:**
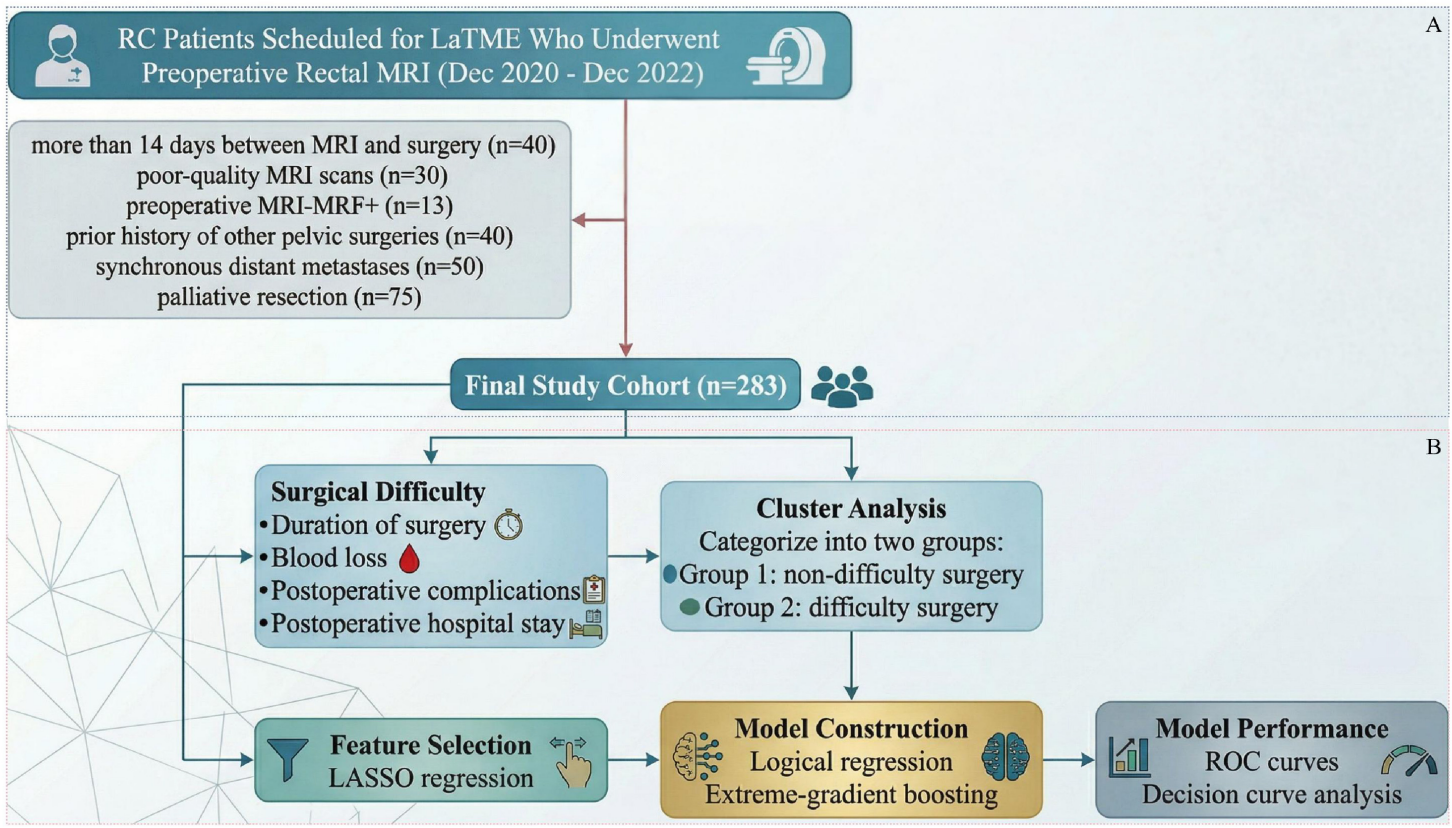
Patient selection flow diagram. **(A)** Inclusion/exclusion flow diagram. **(B)** The study flowchart.

Inclusion criteria were: ➀those with pathologically confirmed rectal adenocarcinoma; ➁LaTME was performed by the same surgical team, under the leadership of a senior surgeon with more than 300 LaTME procedures per year and over 10 years of experience in rectal surgery; ➂complete postoperative pathological and clinical data available in our database; ➃single focal lesion. Exclusion criteria included: ➀a history of prior pelvic surgery other than the current procedure; ➁an interval of more than 14 days between MRI and surgery; ➂suboptimal MRI quality; ➃preoperative MRI-positive circumferential resection margin (MRF+); ➄synchronous distant metastases; ➅palliative resections. The exclusion flow is shown in Figure 1A.

A total of 283 cases were finally included. Patient data were retrospectively obtained from our clinicopathological databases, including gender, age, BMI, carcinoembryonic antigen (CEA), carbohydrate antigen 19–9 (CA19–9), a history of neoadjuvant chemoradiotherapy (nCRT), pathological T stage (pT stage), pathological N stage (pN stage), differentiation, lymphovascular and perineural invasion, tumor budding (TB), tumor deposition, resection margin, and the surgical difficulty indices (including duration of surgery, blood loss, postoperative complications and postoperative hospital stay) (5, 9, 10).

### MRI

Preoperative rectal MRI scans were performed on a 3.0 Tesla MRI system (MAGNETOM Skyra, Siemens Healthcare, Erlangen, Germany) using an 18-channel phased array body coil. A glycerol enema (20 mL) was administered prior to scanning. MRI sequences included sagittal T2-weighted imaging (T2WI), oblique axial high-resolution T2WI, axial diffusion-weighted imaging (DWI) (*b* = 0, 1,000 s/mm^2^), axial T1-weighted imaging (T1WI) and gadolinium contrast-enhanced T1WI (axial, sagittal and coronal). The imaging parameters of the MRI protocol are listed in Supplementary Table S1. The total scan time was approximately 10 min. For patients who underwent nCRT, the post-nCRT MRI were evaluated.

### Image analysis

**FIGURE 2:**
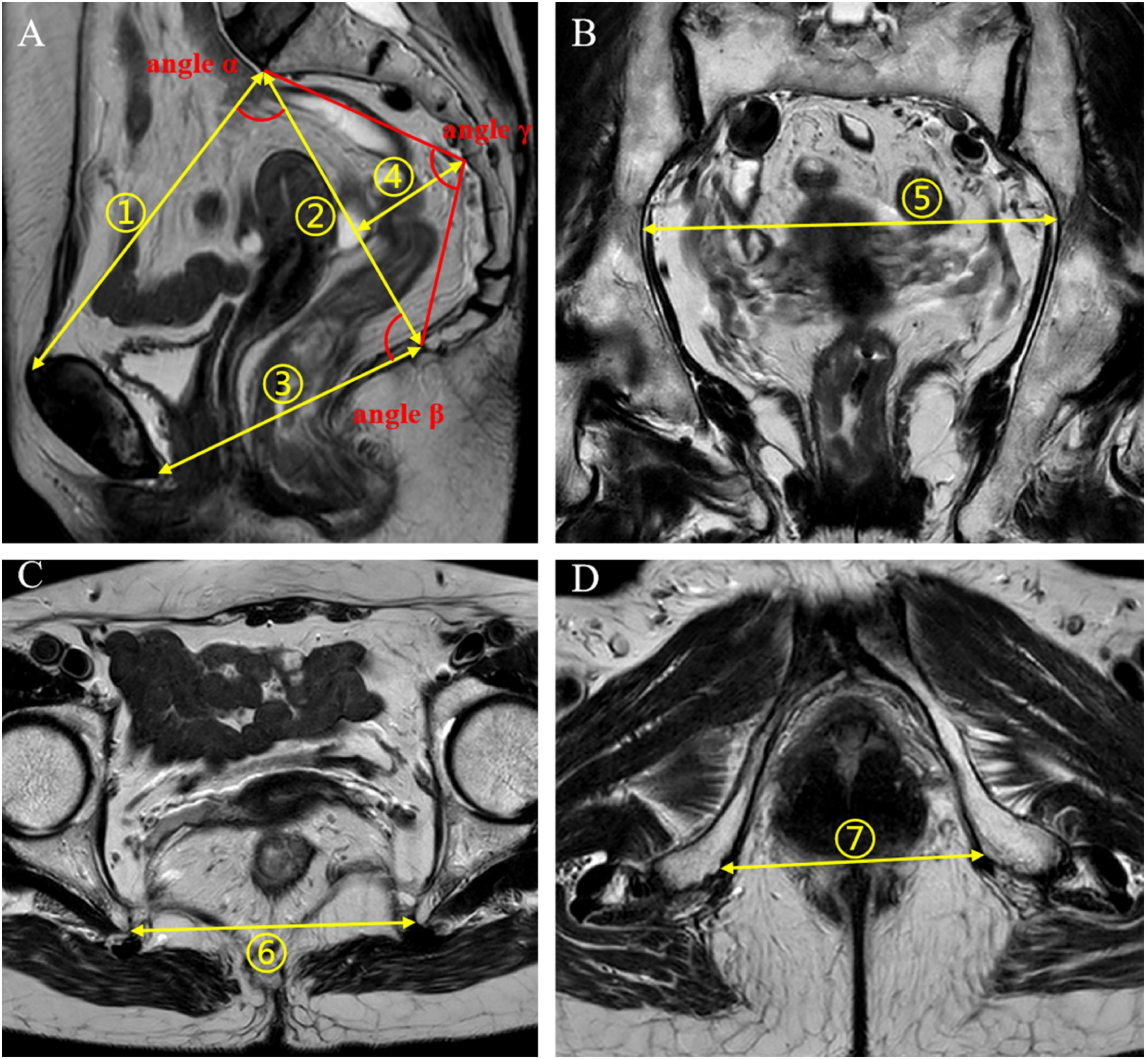
Images showing the MRI-based pelvimetric parameters. **(A)** Sagittal MRI showing ➀the pelvic inlet (distance between sacral promontory and superior aspect of pubic symphysis), ➁the pelvic depth (distance between sacral promontory and tip of the coccyx), ➂the pelvic outlet (distance between tip of the coccyx and inferior aspect of pubic symphysis), and ➃the sacral depth (perpendicular distance between pelvic depth and the deepest portion of the sacrococcygeal hollow) and the pelvic angles (α, β, γ) (α: angle between pelvic inlet and the pelvic depth; β: angle between the pelvic outlet and the pelvic depth; γ: angle between sacral promontory and the tip of the coccyx based on the deepest sacrococcygeal point). **(B)** Coronal MRI showing ➄the transverse diameter (the maximum distance between the bilateral iliopectineal lines). **(C)** Axial MRI showing ➅the interspinous distance (distance between tips of ischial spines). **(D)** Axial MRI showing ➆intertuberous distance (distance between the lowest points of the ischial tuberosities).

Anatomical pelvimetry, including pelvic inlet, pelvic outlet, pelvic depth, sacral depth, angle α, angle β, angle γ, interspinous distance, intertuberous distance, and transverse diameter were independently assessed by two radiologists (SZ and FC, with > 5 years of experience in rectal MRI) on preoperative MRI data. The definition and schematic diagram of each parameter are shown in Figure 2.

**FIGURE 3:**
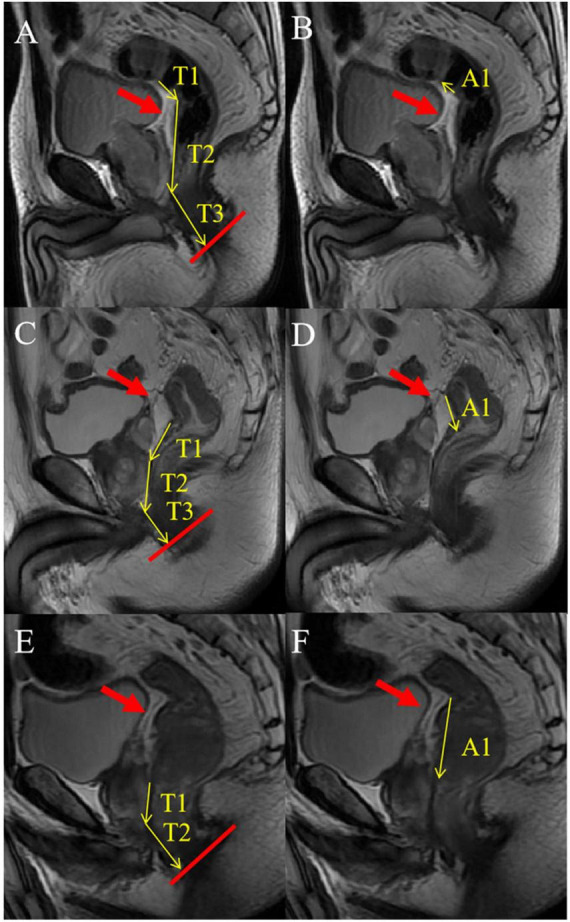
Measurements of tumor height, T-PR and PR height. For tumors above the PR **(A,B)**, tumor height is T1+T2+T3 (A), T-PR is A1 **(B)**, and PR height is A1+T1+T2+T3. A1 is a negative value. For tumors straddling the PR **(C,D)**, tumor height is T1+T2+T3 (C), T-PR is A1 **(D)**, and PR height is A1+T1+T2+T3. A1 is a positive value. For tumors below the PR **(E,F)**, tumor height is T1+T2 **(E)**, T-PR is A1 **(F)**, and PR height is A1+T1+T2. A1 is a positive value. PR, peritoneal reflection (red arrow); T-PR, distance from the inferior tumor margin to PR; above the PR (the distal end of the tumor reaches above the height of the PR) straddling the PR (the distal end of the tumor reaches below the height of the PR and the proximal end of the tumor reaches above the height of the PR) below the PR (the proximal end of the tumor reaches below the height of the PR).

The tumor height, the PR height, the T-PR, and L-PR were assessed on sagittal T2WI. The L-PR was categorized as above, straddling, or below the PR. Tumor height was the distance separating the inferior tumor margin and AV. T-PR was the distance separating the inferior tumor margin and the PR; this distance was negative for lesions above the PR and positive for others (Figure 3). Therefore, the PR height was calculated as tumor height plus T-PR.

These readers were blind to clinicopathological data. We compared the measured values by these two radiologists and used the mean value for each patient in the final analysis. In case of discrepancy in qualitative indices, a third reader (CS with 11 years of experience in rectal MRI) was involved in making the final decision.

### Statistical analysis

The normality of continuous variables was assessed using the one-sample Kolmogorov-Smirnov test. Normally and skewed distributed variables were presented as mean ± standard deviation (SD) and median (quartile); categorical data were presented as percentages. Categorical and continuous data were compared using Chi-square or Fisher’s exact test and independent-samples *t*-test. Hierarchical clustering was used to divide all patients into two groups based on their surgical difficulty indices. The interobserver reproducibility for continuous variables was assessed based on the intraclass correlation coefficient (ICC) and coefficient of variability (CV). ICCs > 0.75, 0.4–0.75, and < 0.4 indicated excellent, good and poor agreement, respectively. Interobserver variability of L-PR was analyzed using the Kappa test. Least absolute shrinkage and selection operator (LASSO) regression and logistic regression (LR) were used to identify factors influencing the operative difficulty of LaTME. LR and the extreme-gradient boosting (XGB) models were constructed. The receiver operating characteristic (ROC) curve and decision curve analysis (DCA) were utilized to compare the performance of the two models. SPSS version 22.0 (SPSS, Chicago, IL, United States) and R version 3.4.3 were used for statistical analyses. *P* < 0.05 was considered statistically significant. Figure 1B presents the study flow.

## Results

### Patient characteristics

A total of 531 RC cases were enrolled from the same surgical team at Changhai Hospital. Following exclusion criteria, 283 participants (204 males and 79 females), aged 60.98 ± 11.17 years (ranging from 26 to 88 years old) were included in the final analysis. All resection margins were negative (including proximal, distal, and circumferential). Only 16 cases (5.7%) experienced Grade I and II surgical complications,, including wound infection (*n* = 4), urinary tract infection (*n* = 1), transient serum creatinine elevation (*n* = 2), pulmonary infection (*n* = 3), intra-abdominal abscess (*n* = 3), and bowel obstruction (*n* = 3). Other detailed patient characteristics are shown in Table 1.

**TABLE 1:** Patient demographics and clinicopathologic findings.

Variable	*N* = 283
Age (years)	60.98 ± 11.17 (26–88)
Gender (M/F)	204/79
BMI (kg/m^2^)	24.00 (21.97, 26.08)
CEA level (%)	
≤ 5 ng/mL	179 (63.3)
> 5 ng/mL	104 (36.7)
CA19–9 level (%)	
≤ 37 U/mL	245 (86.6)
> 37 U/mL	38 (13.4)
The history of nCRT (%)	
Yes	57 (20.1)
No	226 (79.9)
Pathological T staging (%)	
pT0 (pCR)	8 (2.8)
pT1	22 (7.8)
pT2	66 (23.3)
pT3	182 (64.3)
pT4	5 (1.8)
Pathological N staging (%)	
pN0	148 (52.3)
pN1	91 (32.2)
pN2	44 (15.5)
L-PR (%)	
above the PR	74 (26.1)
straddling the PR	114 (40.3)
below the PR	95 (33.6)
Differentiation (%)	
Well	13 (4.6)
Moderate	262 (92.6)
Poor	8 (2.8)
Perineural invasion (%)	
Positive	67 (23.7)
Negative	216 (76.3)
Tumor budding (%)	
Bd 1	239 (84.4)
Bd 2	37 (13.1)
Bd 3	7 (2.5)
Lymphovascular invasion (%)	
Positive	44 (15.5)
Negative	239 (84.5)
Tumor deposit (%)	
Positive	63 (22.3)
Negative	220 (77.7)
Duration of surgery (min)	190 (150, 235)
Blood loss (mL)	100 (50, 200)
Postoperative hospital stay (day)	6 (6, 7)
Postoperative complications (%)	
Yes	16 (5.7)
No	267 (94.3)
Tumor height (cm)	7.60 (6.00, 10.00)
PR height (cm)	9.80 (9.00, 10.50)
T-PR (cm)	2.00 (0.00, 3.60)
Pelvic inlet (cm)	11.65 ± 0.05
Pelvic outlet (cm)	8.00 (7.50,8.60)
Pelvic depth (cm)	12.59 ± 0.06
Sacral depth (cm)	4.00 (3.50,4.40)
Angle α (°)	59.53 ± 0.32
Angle β (°)	77.34 ± 0.38
Angle γ (°)	112.17 ± 0.53
Interspinous distance (cm)	9.50 (8.80,10.30)
Intertuberous distance (cm)	10.55 (9.60,11.70)
Transverse diameter (cm)	12.75 (12.30,13.30)

BMI, body mass index; CEA, carcinoembryonic antigen; CA19–9, carbohydrate antigen 19–9; nCRT, neoadjuvant chemoradiotherapy; pCR, pathological complete response; Bd, budding; PR, anterior peritoneal reflection; T-PR, the distance from the inferior tumor margin to PR; L-PR, the tumor location with regard to PR.

**FIGURE 4:**
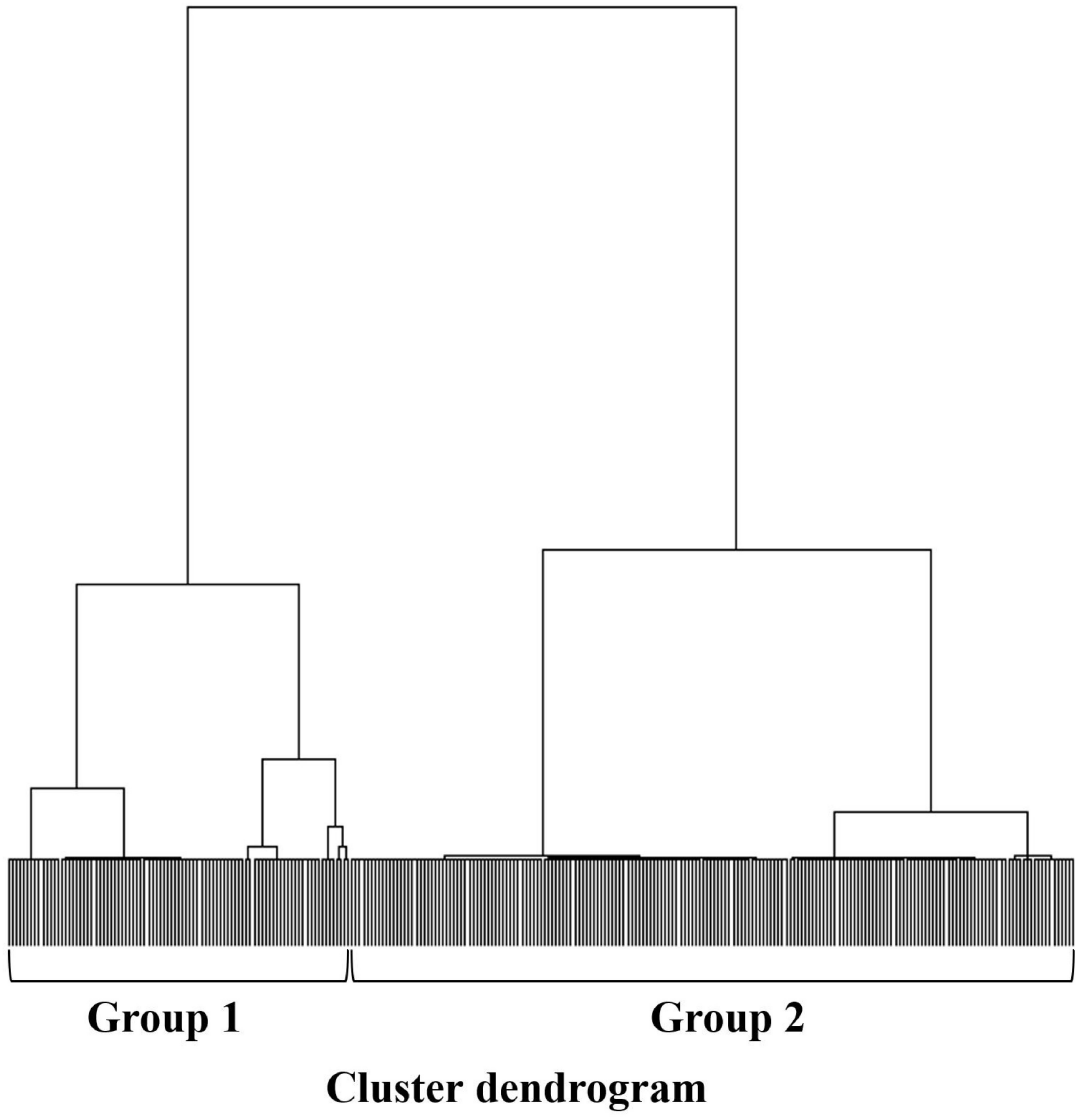
The cluster dendrogram of the LARC patients. Group 1: patients that had a non-difficulty surgery. Group 2: patients that had a difficulty surgery.

Due to the lack of a unified standard and different weighting of each factor to define surgical difficulty, we used hierarchical clustering analysis to comprehensively consider the four indicators and group all patients accordingly (Figure 4). Finally, patients were divided into group 1 (*n* = 71) and group 2 (*n* = 212). The data of the four indicators in Group 2 were higher than those in Group 1 (Table 2), so we defined patients in Group 2 as the surgical difficulty group and patients in Group 1 as the non-difficulty group. The correlation among the surgical indicators is shown in Supplementary Figure S1.

**TABLE 2:** Comparison of evaluating indicators for the operative difficulty of LaTME in patients.

Variable	Group 1 (*n* = 71)	Group 2 (*n* = 212)	*P*-value
Duration of surgery (min)	193.03 ± 63.70	206.52 ± 76.37	0.145
Blood loss (mL)	119.58 ± 70.70	132.08 ± 100.53	0.252
Postoperative hospital stay (day)	6.35 ± 2.09	6.82 ± 3.22	0.160
Postoperative complications			0.074
Yes	1 (1.41)	15 (7.08)	
No	70 (98.59)	197 (92.92)	

Group 1, patients that had a non-difficulty surgery; Group 2, patients that had a difficulty surgery; LaTME, laparoscopic total mesorectal excision.

### Interobserver variability of the measurements

There was excellent reproducibility for the measurement parameters (Table 3), and the Kappa value for the interobserver variability of L-PR was 0.843 (*P* < 0.001).

**TABLE 3:** Interobserver variability of measurement parameters.

Parameters	ICC (95% CI)	CV (%)
Tumor height (cm)	0.959 (0.949–0.968)	6.928
PR height (cm)	0.930 (0.912–0.944)	5.648
T-PR (cm)	0.851 (0.812–0.882)	9.814
Pelvic inlet (cm)	0.893 (0.834–0.932)	2.760
Pelvic outlet (cm)	0.875 (0.801–0.923)	3.431
Pelvic depth (cm)	0.951 (0.920–0.970)	1.864
Sacral depth (cm)	0.854 (0.769–0.909)	5.245
Angle α (°)	0.876 (0.802–0.924)	2.889
Angle β (°)	0.918 (0.867–0.950)	2.826
Angle γ (°)	0.735 (0.624–0.817)	3.477
Interspinous distance (cm)	0.838 (0.745–0.899)	6.567
Intertuberous distance (cm)	0.767 (0.665–0.841)	8.304
Transverse diameter (cm)	0.840 (0.763–0.894)	3.049

PR, peritoneal reflection; CI, confidence interval; CV, coefficient of variation; ICC, intraclass correlation coefficient; T-PR, the distance from the inferior tumor margin to PR.

### Construction of the XGB and LR models

**FIGURE 5:**
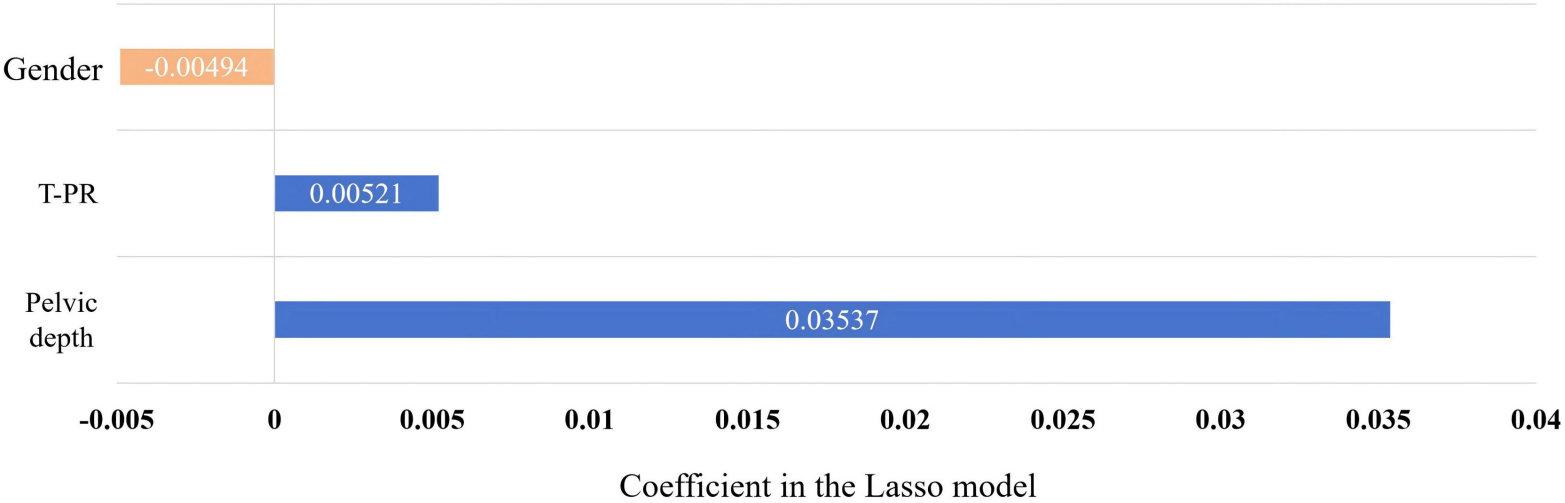
The coefficient of indicators for affecting the operative difficulty of LaTME in the Lasso model.

All the variables were included in the LASSO regression (10-fold cross-validation) to identify the predictors for the surgical difficulty. Pelvic depth, T-PR, and gender were associated with the surgical difficulty level (Figure 5). The correlation among the predictors is shown in Supplementary Figure S2. The predictors significantly differed between the two groups (Supplementary Figure S3). All the predictors were included to build the XGB model (SET params as: booster = gbtree, objective = rank: pairwise, eta = 0.3, gamma = 5, max_depth = 6, min_child_weight = 1, subsample = 1, colsample_bytree = 1). The predicted probability scores in group 1 and group 2 by the XGB model were considered statistically significant, and the relative importance of the three factors is shown in Supplementary Figure S4. Next, univariate logistic regression was performed, and pelvic depth, T-PR, and gender remained predictors of the surgical difficulty level (Supplementary Table S2). An LR model was established based on pelvic depth, T-PR, and gender.

### Comparison of diagnostic performance between the XGB model and LR model

**FIGURE 6:**
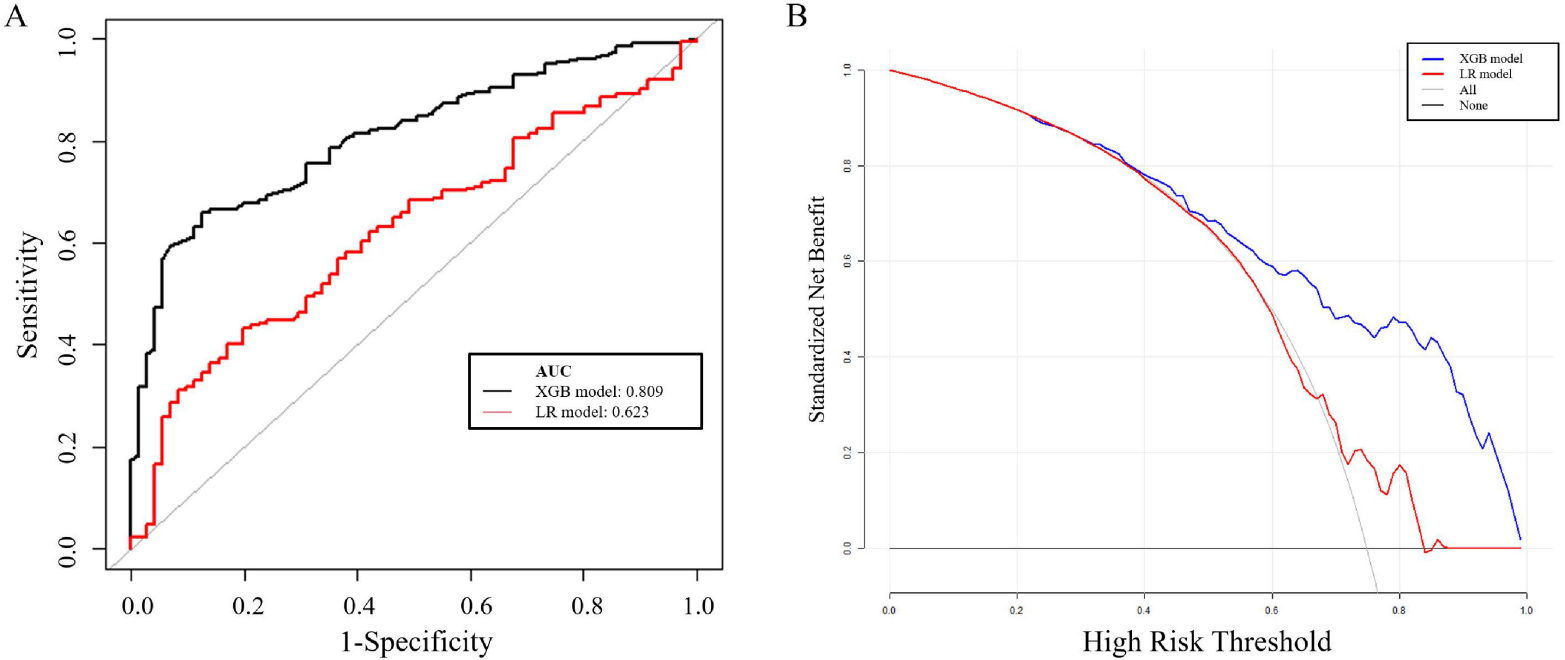
The comparison of ROC **(A)** and DCA **(B)** curves between the XGB Model and LR Model.

In the XGB model, the AUC, sensitivity, specificity, and accuracy were 0.809 (95%CI 0.757–0.862), 0.660, 0.873 and 0.714, respectively. In the LR model, the AUC, sensitivity, specificity, and accuracy were 0.623 (95%CI 0.553–0.694), 0.434, 0.803, and 0.527, respectively. The Delong test showed a statistically significant difference between the two models (Figure 6 and Table 4). DCA confirmed that the XGB model was superior to the LR approach (Figure 6). The confusion matrix of the two models is shown in Supplementary Figure S5. The precision, recall, and F1-score of the predicted classification were 0.940, 0.660, and 0.776, respectively, in the XGB model and 0.868, 0.434, and 0.579, respectively, in the LR model. In addition, an internal validation of the XGB model was performed using a bootstrap resampling method (bootstrap resampling times = 500). The bootstrap algorithm demonstrated satisfactory performance and acceptable calibration. The Hosmer–Lemeshow test yielded a p-value of 0.106, with an AUC of 0.813 (95%CI 0.760–0.862), and sensitivity, specificity, and accuracy of 0.660, 0.873, and 0.714, respectively (Supplementary Figure S6).

**TABLE 4:** The diagnosis performance of two models.

Diagnostic performance metrics	LR model	XGB model
AUC	0.623	0.809
95% CI	0.553–0.694	0.757–0.862
Sensitivity (%)	0.434	0.660
Specificity (%)	0.803	0.873
Accuracy	0.527	0.714
Positive-PV	0.868	0.940
Negative-PV	0.322	0.463
Positive-LR	2.201	5.210
Negative-LR	0.705	0.389
*P*-value	<0.001	

AUC, area under the curve; CI, confidence interval; LR, likelihood ratio; PV, predictive value; LR Model, the logical regression analysis model; XGB Model, the extreme-gradient boosting model.

The proportion of male patients with surgical difficulties was slightly higher. We also conducted a subgroup investigation of gender in the XGB and LR models, and the ROC curve was performed. In the female group, the AUC of the XGB model and LR model were 0.691 (95%CI 0.572–0.811) and 0.545 (95%CI 0.409–0.681), respectively (*P* = 0.044). In the male group, the AUC of the XGB model and LR model were 0.847 (95%CI 0.788–0.906) and 0.642 (95%CI 0.557–0.727), respectively (*P* < 0.001) (Supplementary Figure S7 and Supplementary Table S3).

## Discussion

In this retrospective study, we found that pelvic depth, T-PR, and gender were associated with operative difficulty. The greater the pelvic depth and T-PR, the greater the likelihood of operative difficulty. Also, the proportion of male patients with a difficult surgery was slightly higher than those who underwent a non-difficulty surgery. The XGB model had greater diagnostic efficiency than the LR model. These data may help surgeons in designing the overall surgical plan, which may ultimately help the overall treatment process.

Traditionally, the duration of surgery has been considered one of the main parameters representing technical difficulty during operation (25). However, duration of surgery alone cannot comprehensively reflect all technical difficulties in surgery. Therefore, in this study, duration of surgery plus three clinical indicators were selected to assess surgical difficulty; the three clinical indicators included blood loss, postoperative hospital stay, and postoperative complications (5, 9, 10). Then, based on the literature research, this paper concludes the considerable risk factors for surgical difficulty by clustering them in cluster analysis and then building two surgical difficulty groups system.

Several studies have suggested that surgery is more difficult for males than females (26–28); men have shorter pelvic inlets and outlets and greater pelvic depth than women (8). However, some studies reported opposing results, arguing that gender is not associated with operative difficulty as women have relatively wide and shallow pelvis accompanied by the uterus and bilateral appendages, which affects the operative difficulty (10, 29). Although the relative importance of gender in predicting operative difficulty in the XGB model was relatively low in this study, we found that the proportion of males who underwent a challenging surgery was slightly higher compared to females, which is consistent with the results reported by Lelong et al. (26). However, this could also be related to a higher proportion of males in the present study.

In several studies, pelvimetric parameters have been associated with surgical difficulty, most of which used bony measurements (28, 30). The advantage of measuring bony structures is that they are highly reproducible, as bones can be easily identified on cross-sectional imaging. In the present study, pelvic depth independently predicted the operative difficulty of LaTME in RC patients. Pelvic depth may act as a marker of depth within the pelvis. Deeper locations in the pelvis are likely to be more challenging for the surgeon.

Ogiso and colleagues found that extraperitoneal tumor location is significantly associated with prolonged operative time, which may be because extraperitoneal tumors (tumors below the PR) provide the surgeon with a narrower space for rectal dissection, transection and anastomosis, as the width of the space narrows with increasing pelvic depth for the duration of the LaTME (8). Moreover, Quan et al. reported that tumor distance from the AV is irrelevant to the technical difficulty of rectal surgery (9, 29), while Takashi and his team concluded that shorter tumor height is an independent predictor of longer operative time (31). Although L-PR and tumor height were not independent factors influencing operative difficulty in this study, T-PR resulted as an independent factor (this factor was not considered in previous studies). Its relative importance was comparable to pelvic depth, a significant factor affecting operative difficulty. This may be because T-PR is related to L-PR and tumor height. The higher the T-PR, the lower the tumor location and the greater the surgical difficulty, which was consistent with the results reported by Ogiso et al. (8).

Qin et al. reported that preoperative radiotherapy increases the rate of anastomotic leakage as well as the risk of anastomotic stenosis (32). Furthermore, Veenhof and colleagues found that radiotherapy is associated with operative difficulty (33). However, no history of nCRT was associated with operative difficulty in this study, which may be related to the lower proportion of patients with a history of nCRT treatment. Also, many previous studies have shown that high BMI is an independent predictor of operative time and operative difficulty (8, 26, 31); nonetheless, their results were inconsistent with some other reports (9, 10, 29). BMI may not reflect fat distribution in specific areas of the human body due to race, age, gender, and other factors, which may explain why we found that BMI was not associated with operative difficulty. In order to address this issue, many studies have proposed visceral fat as an even better predictor of operative difficulty than BMI (34–37); thus, we plan to further evaluate this indicator in future studies.

The present study has some limitations. First, operations were carried out by one experienced surgical team to avoid the operator’s influence, which may lead to potential selection bias. This may also explain why many continuous variables studied did not follow a normal distribution. Therefore, prospective randomized studies in multiple centers and larger samples are needed to further confirm these data. Second, previous studies (34–37) have found that baseline medical history (hypertension, diabetes, cardiovascular and cerebrovascular disease, etc.), visceral fat area, volume of the rectum and surrounding soft tissues, and other factors may also affect surgical difficulty but reported inconsistent results. This was a retrospective study, and the data collection was not comprehensive, so more comprehensive prospective studies are required. Third, the definition of surgical difficulty lacks consistent standards. The commonly used indicators (such as operative time, postoperative hospital stay, etc.) differ between different centers, so more objective indicators are needed to evaluate the degree of surgical difficulty, which should also be addressed by future research.

## Conclusion

Preoperative MRI may help to identify patients with high surgical difficulty in LaTME of RC. Pelvic depth, T-PR, and gender were risk factors for operative difficulty. The identification of high-risk patients could be done selectively using more appropriate surgical methods (such as conversion to open surgery, robotic TME, etc.) to improve the quality of surgical resection and achieve a better prognosis.

## Data Availability

The original contributions presented in the study are included in the article/Supplementary material, further inquiries can be directed to the corresponding authors.

## References

[1] Siegel RL Miller KD Jemal A. Cancer statistics, 2020. *CA Cancer J Clin.* (2020) 70:7–30. 10.3322/caac.21590 31912902

[2] Chen W Sun K Zheng R Zeng H Zhang S Xia C et al. Cancer incidence and mortality in China, 2014. *Chin J Cancer Res.* (2018) 30:1–12. 10.21147/j.issn.1000-9604.2018.01.01 29545714 PMC5842223

[3] Conradi LC Rödel C Ghadimi M. Rectal cancer: open questions in 2022 current standards of clinical practice and ongoing trials. *Digestion.* (2022) 103:175–82. 10.1159/000522006 35350020

[4] Chen J Sun Y Chi P Sun B. MRI pelvimetry-based evaluation of surgical difficulty in laparoscopic total mesorectal excision after neoadjuvant chemoradiation for male rectal cancer. *Surg Today.* (2021) 51:1144–51. 10.1007/s00595-020-02211-3 33420827 PMC8215037

[5] Yuan Y Tong D Liu M Lu H Shen F Shi X. An MRI-based pelvimetry nomogram for predicting surgical difficulty of transabdominal resection in patients with middle and low rectal cancer. *Front Oncol.* (2022) 12:882300. 10.3389/fonc.2022.882300 35957878 PMC9357897

[6] Yamamoto S. Comparison of the perioperative outcomes of laparoscopic surgery, robotic surgery, open surgery, and transanal total mesorectal excision for rectal cancer: an overview of systematic reviews. *Ann Gastroenterol Surg.* (2020) 4:628–34. 10.1002/ags3.12385 33319152 PMC7726682

[7] Schietroma M Romano L Apostol AI Vada S Necozione S Carlei F et al. Mid- and low-rectal cancer: laparoscopic vs open treatment-short- and long-term results. Meta-analysis of randomized controlled trials. *Int J Colorectal Dis.* (2022) 37:71–99. 10.1007/s00384-021-04048-9 34716474

[8] Ogiso S Yamaguchi T Hata H Fukuda M Ikai I Yamato T et al. Evaluation of factors affecting the difficulty of laparoscopic anterior resection for rectal cancer: “narrow pelvis” is not a contraindication. *Surg Endosc.* (2011) 25:1907–12. 10.1007/s00464-010-1485-0 21136101

[9] Ma Q Cheng J Bao Y Gao Z Jiang K Wang S et al. Magnetic resonance imaging pelvimetry predicts the technical difficulty of rectal surgery. *Asian J Surg.* (2022) 45:2626–32. 10.1016/j.asjsur.2021.12.022 34961711

[10] Targarona EM Balague C Pernas JC Martinez C Berindoague R Gich I et al. Can we predict immediate outcome after laparoscopic rectal surgery? Multivariate analysis of clinical, anatomic, and pathologic features after 3-dimensional reconstruction of the pelvic anatomy. *Ann Surg.* (2008) 247:642–9. 10.1097/SLA.0b013e3181612c6a 18362627

[11] Haixin Z Qi SU. Predictive value of pelvic anatomical parameters measured by preoperative CT and pathological parameters for the difficulties when performing laparoscopic rectal surgery for mid-low rectal cancer and establishing a scoring system for predicting. *J Clin Pathol Res.* (2019) 39:358–64.

[12] Glynne-Jones R Wyrwicz L Tiret E Brown G Rödel C Cervantes A et al. Rectal cancer: ESMO clinical practice guidelines for diagnosis, treatment and follow-up. *Ann Oncol.* (2017) 28:iv22–40. 10.1093/annonc/mdx224 28881920

[13] Zhang S Chen F Ma X Wang M Yu G Shen F et al. MRI-based nomogram analysis: recognition of anterior peritoneal reflection and its relationship to rectal cancers. *BMC Med Imaging.* (2021) 21:50. 10.1186/s12880-021-00583-7 33731051 PMC7967971

[14] Gao XH Zhai BZ Li J Kabemba JLT Gong HF Bai CG et al. Which definition of upper rectal cancer is optimal in selecting Stage II or III rectal cancer patients to avoid postoperative adjuvant radiation? *Front Oncol.* (2020) 10:625459. 10.3389/fonc.2020.625459 33643920 PMC7907590

[15] Najarian MM Belzer GE Cogbill TH Mathiason MA. Determination of the peritoneal reflection using intraoperative proctoscopy. *Dis Colon Rectum.* (2004) 47:2080–5. 10.1007/s10350-004-0740-7 15657658

[16] Benson AB Venook AP Al-Hawary MM Arain MA Chen YJ Ciombor KK et al. Small bowel adenocarcinoma, version 1.2020, NCCN clinical practice guidelines in oncology. *J Natl Compr Canc Netw.* (2019) 17:1109–33. 10.6004/jnccn.2019.0043 31487687 PMC10191182

[17] Park JS Sakai Y Simon NSM Law WL Kim HR Oh JH et al. Long-Term survival and local relapse following surgery without radiotherapy for locally advanced upper rectal cancer: an international multi-institutional study. *Medicine.* (2016) 95:e2990. 10.1097/MD.0000000000002990 27258487 PMC4900695

[18] Trebeschi S van Griethuysen JJM Lambregts DMJ Lahaye MJ Parmar C Bakers FCH et al. Deep learning for fully-automated localization and segmentation of rectal cancer on multiparametric MR. *Sci Rep.* (2017) 7:5301. 10.1038/s41598-017-05728-9 28706185 PMC5509680

[19] Lu H Yuan Y Zhou Z Ma X Shen F Xia Y et al. Assessment of MRI-Based radiomics in preoperative T staging of rectal cancer: comparison between minimum and maximum delineation methods. *Biomed Res Int.* (2021) 2021:5566885. 10.1155/2021/5566885 34337027 PMC8289571

[20] Kasai S Shiomi A Kagawa H Hino H Manabe S Yamaoka Y et al. The effectiveness of machine learning in predicting lateral lymph node metastasis from lower rectal cancer: a single center development and validation study. *Ann Gastroenterol Surg.* (2022) 6:92–100. 10.1002/ags3.12504 35106419 PMC8786681

[21] Chen X Wang W Chen J Xu L He X Lan P et al. Predicting pathologic complete response in locally advanced rectal cancer patients after neoadjuvant therapy: a machine learning model using XGBoost. *Int J Colorectal Dis.* (2022) 37:1621–34. 10.1007/s00384-022-04157-z 35704090 PMC9262764

[22] Cui Y Yang W Ren J Li D Du X Zhang J et al. Prognostic value of multiparametric MRI-based radiomics model: potential role for chemotherapeutic benefits in locally advanced rectal cancer. *Radiother Oncol.* (2021) 154:161–9. 10.1016/j.radonc.2020.09.039 32976874

[23] Torlay L Perrone-Bertolotti M Thomas E Baciu M. Machine learning-XGBoost analysis of language networks to classify patients with epilepsy. *Brain Inform.* (2017) 4:159–69. 10.1007/s40708-017-0065-7 28434153 PMC5563301

[24] Liu L Yu Y Fei Z Li M Wu FX Li HD et al. An interpretable boosting model to predict side effects of analgesics for osteoarthritis. *BMC Syst Biol.* (2018) 12:105. 10.1186/s12918-018-0624-4 30463545 PMC6249730

[25] Lee JM Han YD Cho MS Hur H Min BS Lee KY et al. Prediction of transabdominal total mesorectal excision difficulty according to the angle of pelvic floor muscle. *Surg Endosc.* (2020) 34:3043–50. 10.1007/s00464-019-07102-4 31482361

[26] Lelong B Bege T Esterni B Guiramand J Turrini O Moutardier V et al. Short-term outcome after laparoscopic or open restorative mesorectal excision for rectal cancer: a comparative cohort study. *Dis Colon Rectum.* (2007) 50:176–83. 10.1007/s10350-006-0751-7 17180257

[27] Hidalgo JM Targarona EM Martinez C Hernandez P Balague C Trias M. Laparoscopic rectal surgery: does immediate outcome differ in respect to sex? *Dis Colon Rectum.* (2010) 53:438–44. 10.1007/DCR.0b013e3181bdbaa7 20305444

[28] Atasoy G Arslan NC Elibol FD Sagol O Obuz F Sokmen S. Magnetic resonance-based pelvimetry and tumor volumetry can predict surgical difficulty and oncologic outcome in locally advanced mid-low rectal cancer. *Surg Today.* (2018) 48:1040–51. 10.1007/s00595-018-1690-3 29961173

[29] Chau J Solomon J Liberman AS Charlebois P Stein B Lee L. Pelvic dimensions on preoperative imaging can identify poor-quality resections after laparoscopic low anterior resection for mid- and low rectal cancer. *Surg Endosc.* (2020) 34:4609–15. 10.1007/s00464-019-07209-8 31620910

[30] de’Angelis N Pigneur F Martínez-Pérez A Vitali GC Landi F Torres-Sánchez T et al. Predictors of surgical outcomes and survival in rectal cancer patients undergoing laparoscopic total mesorectal excision after neoadjuvant chemoradiation therapy: the interest of pelvimetry and restaging magnetic resonance imaging studies. *Oncotarget.* (2018) 9:25315–31. 10.18632/oncotarget.25431 29861874 PMC5982752

[31] Akiyoshi T Watanabe T Ueno M. Pelvic dimensions as a predictor of difficulty in laparoscopic surgery for rectal cancer. *Surg Endosc.* (2011) 25:3122–3. 10.1007/s00464-011-1649-6 21424192

[32] Qin Q Zhu Y Wu P Fan X Huang Y Huang B et al. Radiation-induced injury on surgical margins: a clue to anastomotic leakage after rectal-cancer resection with neoadjuvant chemoradiotherapy? *Gastroenterol Rep.* (2019) 7:98–106. 10.1093/gastro/goy042 30976422 PMC6454846

[33] Veenhof AA Engel AF van der Peet DL Sietses C Meijerink WJ de Lange-de Klerk ES et al. Technical difficulty grade score for the laparoscopic approach of rectal cancer: a single institution pilot study. *Int J Colorectal Dis.* (2008) 23:469–75. 10.1007/s00384-007-0433-5 18185936 PMC2668628

[34] Ozato N Saito S Yamaguchi T Katashima M Tokuda I Sawada K et al. Association between nutrients and visceral fat in healthy Japanese adults: a 2-Year longitudinal study brief title: micronutrients associated with visceral fat accumulation. *Nutrients.* (2019) 11:2698. 10.3390/nu11112698 31703461 PMC6893766

[35] Lee KH Kang BK Ahn BK. Higher visceral fat area/subcutaneous fat area ratio measured by computed tomography is associated with recurrence and poor survival in patients with mid and low rectal cancers. *Int J Colorectal Dis.* (2018) 33:1303–7. 10.1007/s00384-018-3065-z 29713823

[36] Chen B Zhang Y Zhao S Yang T Wu Q Jin C et al. The impact of general/visceral obesity on completion of mesorectum and perioperative outcomes of laparoscopic TME for rectal cancer: a STARD-compliant article. *Medicine.* (2016) 95:e4462. 10.1097/MD.0000000000004462 27603340 PMC5023862

[37] Seki Y Ohue M Sekimoto M Takiguchi S Takemasa I Ikeda M et al. Evaluation of the technical difficulty performing laparoscopic resection of a rectosigmoid carcinoma: visceral fat reflects technical difficulty more accurately than body mass index. *Surg Endosc.* (2007) 21:929–34. 10.1007/s00464-006-9084-9 17285393

