# Supplementary material for: Machine learning for predicting surgical difficulty of laparoscopic total mesorectal excision for rectal cancer: integrating MR-based pelvimetry and peritoneal reflection
Source: Front Med (Lausanne). 2026 Jun 12;13:1853753. doi: 10.3389/fmed.2026.1853753 (PMC13303118; doi:10.3389/fmed.2026.1853753)
Supplement: Supplementary file 1 [file Data_Sheet_1.pdf]

Table S1. Imaging parameters of the MRI protocol

Parameters	Sagittal T2WI	Oblique axial high-resolution T2WI	Axial T1WI	Axial DWI	Axial contrast-enhanced T1WI	Sagittal contrast-enhanced T1WI	Coronal contrast-enhanced T1WI
TR (ms)	5000	4000	3.77	6300	5.9	4.13	3.95
TE (ms)	106	108	1.51	89	2.46	1.92	1.81
FOV (mm^2)	230×230	180×180	300×300	380×380	300×300	300×300	350×350
Matrix	256×256	320×320	246×246	150×150	201×201	240×240	240×240
Number of slices	23	28	60	20	52	56	52
Gap (mm)	1	0	0.8	1	0.9	0.6	0.6
Acceleration factor	2	3	2	2	2	3	3
Bandwidth (Hz/pixel)	200	108	390	2084	390	500	500
Acquisition time (s)	157	250	16	90	16	15	17
Slice thickness (mm)	5	3	3	5	4	3	3

TR: repetition time; TE: echo time; FOV: field of view; DWI: diffusion-weighted imaging, *b* values (averages) = 0 and 1000 s/mm^2

T1WI: T1-weighted imaging; T2WI: T2-weighted imaging

Table S2. Logistic regression analyses of factors that affect the operative difficulty of LaTME in patients

Variables	Univariate logistic regression		Multivariate logistic regression	
	OR (95%CI)	*P* value	OR (95%CI)	*P* value
Pelvic inlet (cm)	0.094 (-0.003,0.190)	0.058		
Pelvic outlet (cm)	0.070 (-0.046, 0.187)	0.238		
Pelvic depth (cm)	0.141 (0.059, 0.223)	0.001	1.339 (1.047,1.713)	0.020
Sacral depth (cm)	-0.087 (-0.265, 0.091)	0.339		
angle α (°)	-0.009 (-0.025, 0.007)	0.250		
angle β (°)	-0.010 (-0.024, 0.004)	0.152		
angle γ (°)	0.009 (-0.001, 0.020)	0.091		
Interspinous distance (cm)	-0.046 (-0.136, 0.044)	0.318		
Intertuberous distance (cm)	-0.020 (-0.086, 0.047)	0.559		
Transverse diameter (cm)	0.003 (-0.122, 0.129)	0.959		
L-PR	-0.179 (-0.408, 0.051)	0.128		
BMI (kg/m^2)	0.023 (-0.006, 0.052)	0.119		
Gender (M/F)	0.248 (0.026, 0.471)	0.030	1.061 (0.567,1.980)	0.852
The history of nCRT	0.210 (-0.040, 0.460)	0.101		
Age (years)	-0.002 (-0.011, 0.007)	0.638		
T-PR (cm)	0.043 (0.002, 0.084)	0.042	1.094 (0.979,1.222)	0.112
Tumor height (cm)	-0.028 (-0.065, 0.010)	0.147		
PR height (cm)	0.039 (-0.046, 0.123)	0.371		

LaTME: laparoscopic total mesorectal excision

Table S3. The diagnosis performance of XGB model and LR model in male and female

	**XGB Model Female**	**LR Model Female**	**XGB Model Male**	**LR Model Male**
AUC	0.691	0.545	0.847	0.642
95%CI	0.572-0.811	0.409-0.681	0.788-0.906	0.557-0.727
Sensitivity (%)	0.411	0.232	0.756	0.500
Specificity (%)	0.957	0.957	0.833	0.750
Accuracy	0.570	0.443	0.775	0.559
Positive-PV	0.958	0.929	0.937	0.867
Negative-PV	0.400	0.339	0.513	0.316
Positive-LR	9.446	5.339	4.539	2.000
Negative-LR	0.616	0.803	0.292	0.667
P value	0.044		<0.001	

AUC: area under the curve. CI: confidence interval; LR: likelihood ratio; PV: predictive value; LR Model: the logical regression analysis model; XGB Model: the extreme-gradient boosting model

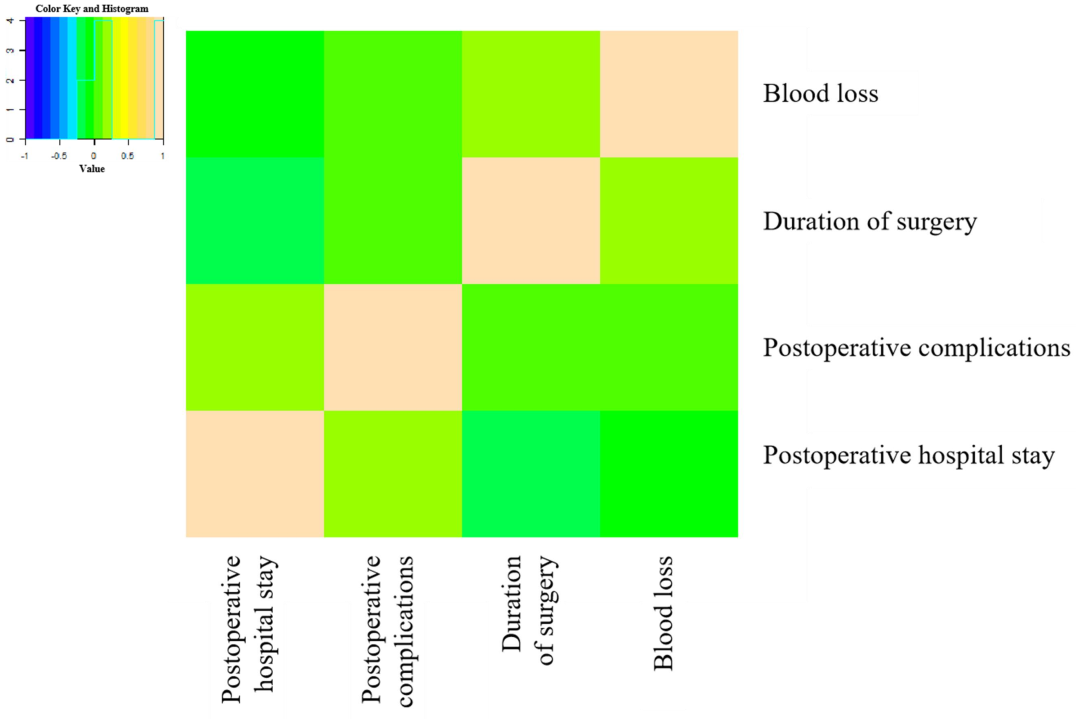

Figure S1. The correlation between indicators for evaluating the operative difficulty of LaTME.

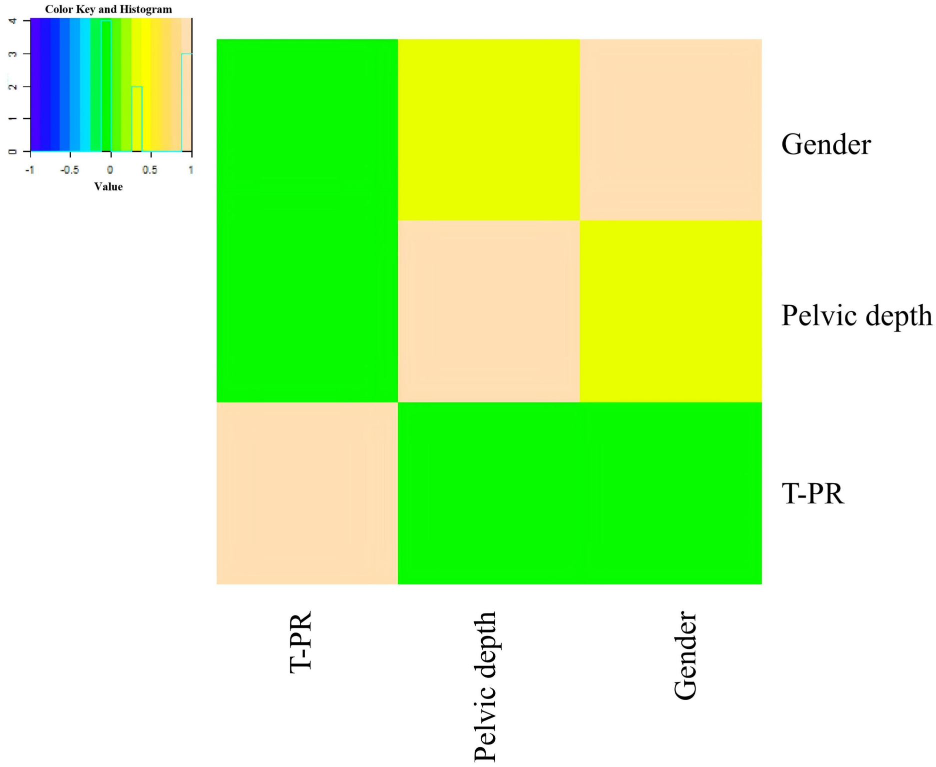

Figure S2. The correlation among the predictors for affecting the operative difficulty of LaTME.

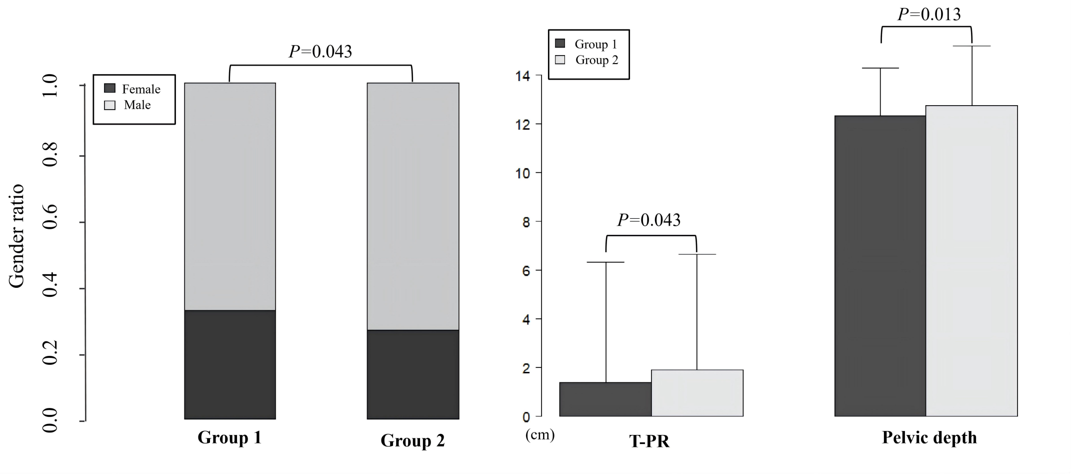

Figure S3. Histogram of **(A)** gender ratio and **(B)** T-PR and pelvic depth in group 1 and 2.

Group 1: patients that had a non-difficulty surgery.

Group 2: patients that had a difficulty surgery.

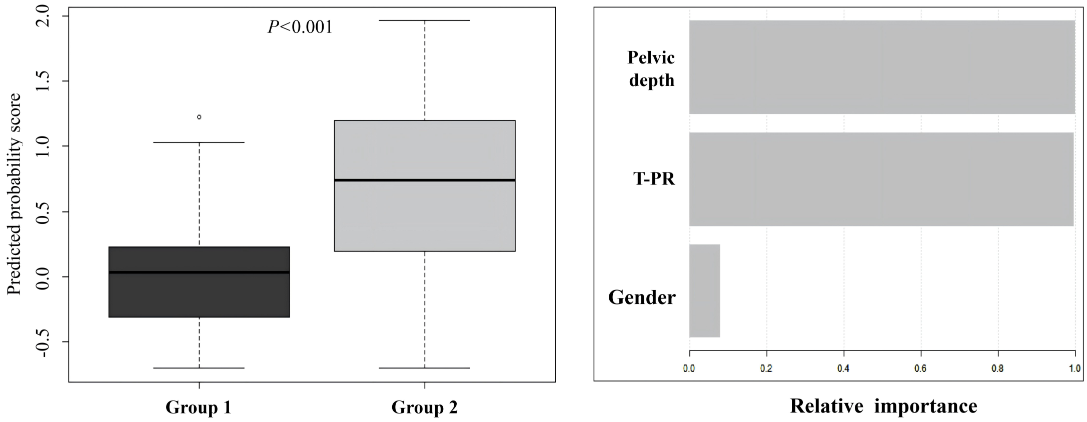

Figure S4. (A) Box plots of predicted probability scores in group 1 and group 2 by the XGB model. **(B)** The relative importance of pelvic depth, T-PR height, and gender in the XGB model.

Group 1: patients that had a non-difficulty surgery.

Group 2: patients that had a difficulty surgery.

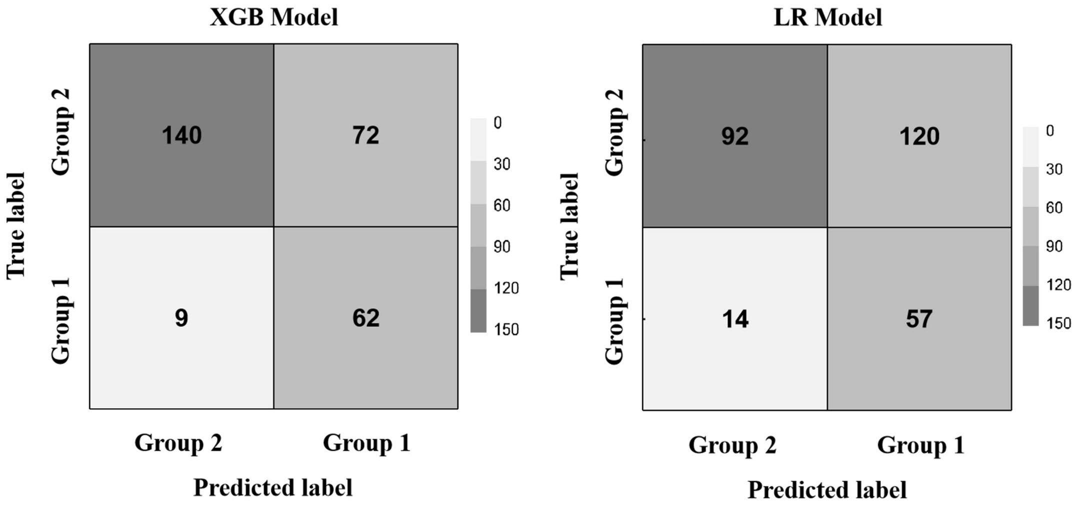

Figure S5. The confusion matrix of the XGB model and LR Model.

Group 1: patients that had a non-difficulty surgery.

Group 2: patients that had a difficulty surgery.

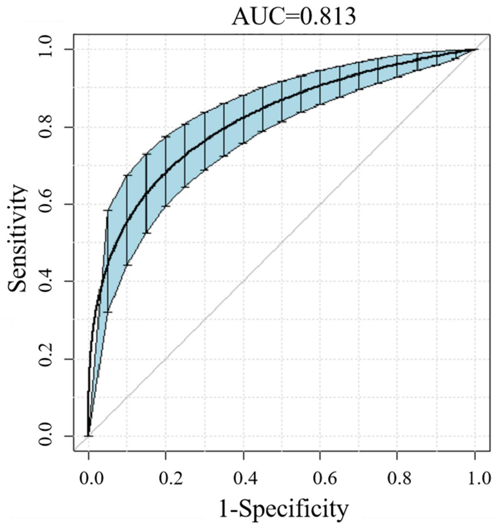

Figure S6. The ROC of the continuous predictor.

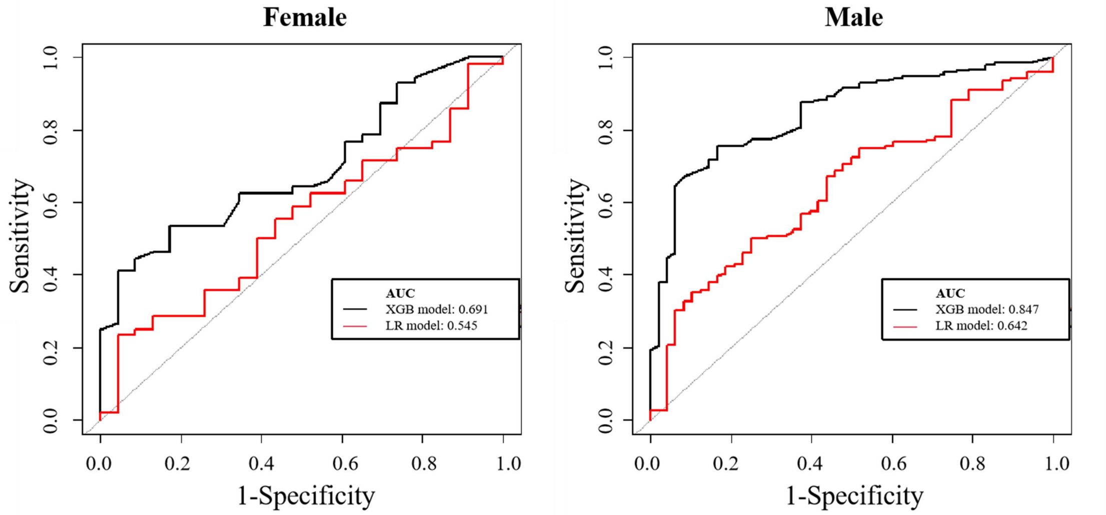

Figure S7. The ROC curves of the two models in females and males.
